# Real-World Integration of an Automated Tool for Intracranial Hemorrhage Detection in an Unselected Cohort of Emergency Department Patients—An External Validation Study

**DOI:** 10.3390/diagnostics16020282

**Published:** 2026-01-16

**Authors:** Ronald Antulov, Martin Weber Kusk, Gustav Højrup Knudsen, Sune Eisner Lynggaard, Simon Lysdahlgaard, Vladimir Antonov

**Affiliations:** 1 Department of Radiology and Nuclear Medicine, Esbjerg and Grindsted Hospitals, University Hospital of Southern Denmark, 6700 Esbjerg, Denmark; 2 Department of Regional Health Research, University of Southern Denmark, 5230 Odense, Denmark; 3 Department of Emergency Medicine, Esbjerg and Grindsted Hospitals, University Hospital of Southern Denmark, 6700 Esbjerg, Denmark

**Keywords:** RAPID, non-contrast head computed tomography, intracranial hemorrhage, deep learning, generalizability

## Abstract

**Background/Objectives**: Intracranial hemorrhage (ICH) is a life-threatening condition that can be rapidly detected by non-contrast head computed tomography (NCCT). RAPID ICH is a deep learning (DL) tool for automatic ICH identification using NCCT. Our aim was to assess the real-world performance of RAPID ICH compared to that of a first-year radiology resident on consecutively acquired NCCTs from patients referred from the Emergency Department. **Methods**: This single-center retrospective cohort study included NCCTs acquired on the same CT scanner over three months. Exclusion criteria were motion or metallic artifacts that substantially degraded the NCCT quality and incomplete NCCTs. Two senior neuroradiologists conducted ground-truth labeling of the NCCTs regarding ICH presence in a binary manner. The first-year radiology resident assessed NCCTs for ICH presence and was blinded to the ground-truth labeling. Sensitivity, specificity, positive predictive value (PPV), and negative predictive value (NPV) were computed for the RAPID ICH identifications and for the first-year radiology resident’s ICH identifications. **Results**: After applying exclusion criteria, 844 NCCTs remained. Ground-truth labeling found ICH in 63 NCCTs. RAPID ICH showed 87.3% sensitivity, 74% specificity, 21.3% PPV, and 98.6% NPV, while the first-year radiology resident achieved 95.2% sensitivity, 90.8% specificity, 45.5% PPV, and 99.6% NPV. There were 8 false-negative and 203 false-positive RAPID ICH identifications. **Conclusions**: RAPID ICH’s sensitivity and specificity were lower than in prior studies performed using RAPID ICH, and there was a high number of false-positive RAPID ICH identifications, limiting the generalizability of the assessed version of this DL tool. Testing DL tools by comparing them with radiologists of varying experience can provide valuable insights into their performance.

## 1. Introduction

Non-contrast head computed tomography (NCCT) remains the mainstay of diagnosis and treatment decision-making for patients with suspected intracranial hemorrhage (ICH) in emergency settings [1]. Prompt ICH identification assists in determining patients who are suitable for surgical intervention, while exclusion of ICH in patients with suspected stroke can help assess their eligibility for thrombolysis [2,3]. Given the vital importance of NCCT in detecting ICH, it is essential to ensure the round-the-clock availability of in-house radiological services. Interpretation of NCCTs outside regular working hours is often assigned to radiology residents or junior radiologists, comprising approximately half of all after-hours CT examinations [4,5]. Previous studies have demonstrated discrepancies in the interpretation of NCCTs performed after-hours between radiology residents and neuroradiologists, where the incorrect identification of ICH accounted for 0.4% of all included NCCTs in a study by Psychogios et al. and 0.6% of all included NCCTs in a study by Strub et al. [6,7]. In recent years, automated tools have been developed to assist radiologists in detecting ICH on NCCTs and streamline their workflow.

Artificial intelligence (AI) technologies have significantly improved efficiency in emergency radiology by reducing report turnaround times and prioritizing critical findings, such as, for example, ICH, as well as allowing radiologists to focus on complex examinations [8]. Several Food and Drug Administration (FDA)-approved and “Conformité Européene” (CE)-marked automated tools for ICH detection based on deep learning (DL)—a branch of machine learning (ML) and AI—like RAPID ICH (https://www.rapidai.com), Viz Hemorrhage (www.viz.ai), Aidoc (www.aidoc.com), or Brainomix 360 Triage Stroke (www.brainomix.com) are commercially available. The commercial nature of these tools restricts the full disclosure of the training of the ML model and the training datasets used. Therefore, the validation of commercially available automated tools for ICH detection when implemented into different real-world clinical settings is a recognized challenge [9,10,11].

The present study constitutes a retrospective external validation of RAPID ICH, a tool integrated within the RAPID software platform (iSchemaView, Menlo Park, CA, USA), which is utilized for processing neuroimaging datasets of suspected stroke patients. RAPID ICH is a DL convolutional neural network-based tool developed to automatically identify ICH on NCCT images. Prior validation studies of RAPID ICH have shown high sensitivity and specificity in ICH detection [12,13].

Our aim was to assess the real-world performance of RAPID ICH in comparison to a first-year radiology resident on a collection of consecutively acquired NCCTs referred from the Emergency Department (ED) over a period of three months.

## 2. Materials and Methods

### 2.1. Study Population

The study included ED NCCTs performed at the Esbjerg and Grindsted Hospitals—University Hospital of Southern Denmark on adult patients between July 2021 and September 2021. Patients had different clinical indications, including head trauma, suspected stroke or intracranial bleeding, and dizziness. Repeated NCCTs of the same patient, if performed at different time points, were also included. Exclusion criteria were severe motion or metallic artifacts that substantially degraded NCCT quality and incomplete NCCTs.

### 2.2. Definition of ICH

ICH is identified as an intracranial hyperdense finding of over 60 Hounsfield units, irrespective of size, without an alternative explanation [14]. The classification of ICHs was performed using the following categories: epidural/subdural, subarachnoid, intraparenchymal, intraventricular, and combined, if two or more types of ICHs were present.

### 2.3. Image Acquisition

All NCCTs were acquired on the same CT scanner (Somatom Force, Siemens Healthcare GmbH, Forchheim, Germany) with the following parameters: tube voltage of 120 kV, single collimation width of 0.6 mm, reconstructed slice thickness of 5 mm, reconstruction method of Advanced Modeled Iterative Reconstruction (ADMIRE) level 3, and reconstruction kernel of HR32. Only 5 mm reconstructed axial images were used for further evaluation and were automatically sent to RAPID ICH.

### 2.4. AI Software—RAPID ICH

Training for the RAPID ICH ML model included a wide variety of pathologic features, image properties, and characteristics collected from hospitals in the USA and Australia for patients screened between 2008 and 2018, which enabled the sampling of a broad range of imaging protocols, scanner models, and commonly encountered acquisition artifacts; the ground truth for training was determined by an experienced neuroradiologist [15]. The FDA clearance for the latest version of RAPID ICH (version 3.0) reported a sensitivity of 96.8% and specificity of 100% in identifying NCCT images containing an ICH, which represents an improvement compared to the previous RAPID ICH (version 1.0), for which a sensitivity of 89.9% and specificity of 94.3% were stated [16,17]. RAPID ICH identifies suspected ICHs as small as 0.4 mL [18]. RAPID ICH (version 1.0) was used in this study, since it was the current version during the research period. The axial images where there was a suspected ICH were sent back to the hospital’s picture archiving and communication systems (PACS) and marked as “suspected hemorrhage”.

### 2.5. Data Collection

All collected NCCTs were evaluated for the presence of exclusion criteria by a second-year radiology resident (S.E.L.). The remaining NCCTs were retrieved from the hospital’s PACS and checked for the presence of axial images that RAPID ICH identified as “suspected hemorrhage”. When such images were found, the corresponding NCCT was considered ICH-positive by RAPID ICH, and when they were not, the NCCT was considered ICH-negative by RAPID ICH. The 5 mm reconstructed axial images of all remaining NCCTs were anonymized, transferred to an external PACS system, and prepared for further radiological assessment.

### 2.6. Radiological Assessment

Two senior neuroradiologists (R.A. and V.A.) performed ground-truth labeling of the anonymized 5 mm reconstructed axial images of all remaining NCCTs regarding ICH presence in a binary manner—present ICH or absent ICH—and if ICH was detected, the type of ICH, as previously described, had to be classified. Discrepancies between the two senior neuroradiologists were solved by consensus. The first-year radiology resident (G.K.), who had completed a 3-month-long neuroradiology training, evaluated the anonymized 5 mm reconstructed axial images of all remaining NCCTs for ICH presence in a binary manner (present ICH or absent ICH). All radiologists controlled the window width and levels for all images and were unaware of RAPID ICH identifications. The first-year radiology resident was not previously involved with the evaluation of the NCCTs included in the study, was blinded to the ground-truth labeling made by the two senior neuroradiologists, and the evaluation took place during regular working hours to simulate an everyday work environment. All NCCTs that, after RAPID ICH identification, disagreed in comparison to ground-truth labeling, corresponding to false-negative (FN) RAPID ICH identifications and false-positive (FP) RAPID ICH identifications, were analyzed and categorized by the same two senior neuroradiologists.

### 2.7. Statistical Analysis

Descriptive statistics included the median, absolute numbers, and the relative proportions (%). The inter-reader agreement between the two senior neuroradiologists for ground-truth labeling regarding the presence of ICH was assessed by Cohen’s kappa with a 95% confidence interval (CI). Sensitivity, specificity, positive predictive value (PPV), negative predictive value (NPV), and area under the curve (AUC) with a 95% CI, as well as the F1 score, were computed for the RAPID ICH identifications against the ground-truth labeling, as well as for the first-year radiology resident’s ICH identifications against the ground-truth labeling. The homogeneity of odds ratios across the contingency tables corresponding to RAPID ICH identifications and the first-year radiology resident’s ICH identifications was examined using the Breslow–Day test. Conditional independence between the RAPID ICH identifications and the first-year radiology resident’s ICH identifications was assessed with the Cochran–Mantel–Haenszel test. Statistical analyses were performed using IBM SPSS Statistics for Windows, version 26 (IBM Corp., Armonk, NY, USA).

## 3. Results

### 3.1. Descriptive Characteristics

**Figure 1:**
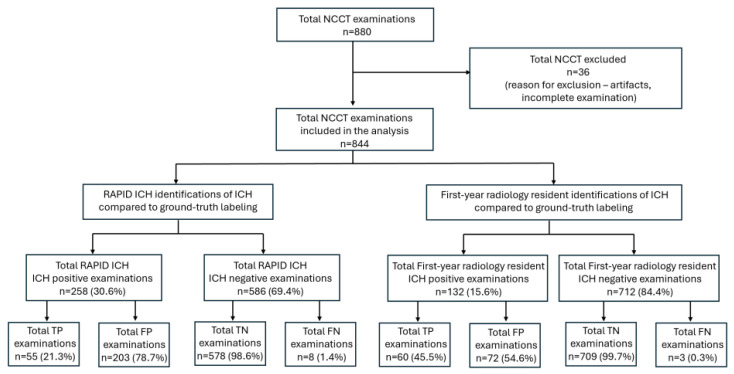
Overview of the study showing the number of included non-contrast head computed tomography (NCCT) examinations and the number of positive and negative intracranial hemorrhage (ICH) examinations according to RAPID ICH and the first-year radiology resident, as well as subgroup evaluations of the true-positive (TP), false-positive (FP), true-negative (TN), and false-negative (FN) identifications by RAPID ICH and the first-year radiology resident compared to ground-truth labeling.

During the study period, a total of 880 consecutive NCCT examinations from the ED were acquired. After applying the exclusion criteria, 36 NCCT examinations were removed, leaving 844 NCCT examinations for further assessment (Figure 1). Those NCCT examinations were derived from a cohort of 821 patients that included 463 (56.4%) male patients and 358 (43.6%) female patients. The median age of the cohort was 67.3 years.

### 3.2. Ground-Truth Labeling

Ground-truth labeling regarding the presence of ICH identified 63 (7.5%) NCCTs with an ICH. The two senior neuroradiologists agreed on 842 of the 844 assessments, and Cohen’s kappa was calculated to be 0.98 [95% CI: 0.96–1]. The classifications of the ICHs were as follows: 14 (22.2%) epidural/subdural, 8 (12.7%) subarachnoid, 34 (53.9%) intraparenchymal, 2 (3.2%) intraventricular, and 3 (4.7%) combined. The two senior neuroradiologists matched on 57 of the 63 ICH classifications.

### 3.3. Analysis of RAPID ICH Identifications and First-Year Radiology Resident ICH Identifications

When RAPID ICH identifications were compared with ground-truth labeling, there were 55 correctly identified ICH-positive NCCTs and 578 correctly identified ICH-negative NCCTs. The sensitivity of RAPID ICH was 87.3% [95% CI: 76.5–94.4%], its specificity was 74% [95% CI: 70.8–77.1%], its PPV was 21.3% [95% CI: 16.5–26.8%], its NPV was 98.6% [95% CI: 97.3–99.4%], and its AUC 0.81 [95% CI: 0.76–0.85]. The F1 score was 0.34 (Table 1).

When the first-year radiology resident’s ICH identifications were compared with ground-truth labeling, there were 60 correctly identified ICH-positive NCCTs and 709 correctly identified ICH-negative NCCTs. The first-year radiology resident’s sensitivity was 95.2% [95% CI: 86.7–99%], their specificity was 90.8% [95% CI: 88.5–92.7%], their PPV was 45.5% [95% CI: 36.8–54.3%], their NPV was 99.6% [95% CI: 98.8–99.9%] and their AUC 0.93 [95% CI: 0.90–0.96]. Their F1 score was 0.62 (Table 1).

A comparison of contingency tables, evaluated against ground-truth labeling, revealed a highly significant difference (*p* < 0.001) in the diagnostic accuracies of ICH identification between RAPID ICH and the first-year radiology resident.

### 3.4. Discrepancies Between RAPID ICH Identifications and Ground-Truth Labeling

**Figure 2:**
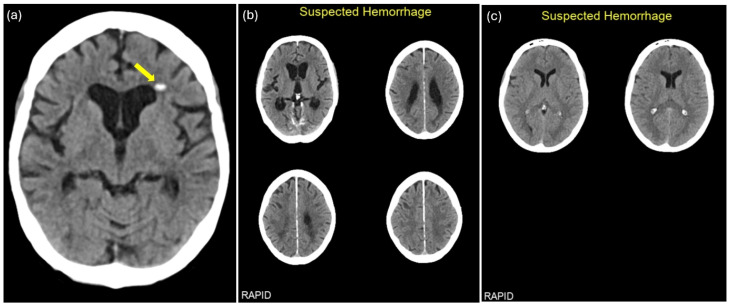
Examples of discrepancies between RAPID ICH identifications and ground-truth labeling presented on 5 mm reconstructed axial non-contrast head computed tomography (NCCT) images. (**a**) Example of a false-negative RAPID ICH identification where a small intraparenchymal hemorrhage (yellow arrow) in the left frontal lobe was not recognized; (**b**) example of a false-positive RAPID ICH identification probably related to dural thickening; (**c**) example of a false-positive RAPID ICH identification probably related to choroid plexus calcifications.

There were eight FN RAPID ICH identifications, of which four (50%) missed acute intraparenchymal hemorrhages, two (25%) were acute epidural/subdural hemorrhages, one (12.5%) was a fresh subarachnoid hemorrhage, and one (12.5%) was a subacute intraparenchymal hemorrhage. The missed ICHs were of sizes up to 6 mm (Figure 2a).

There were 203 FP RAPID ICH identifications, of which most could be explained by an incorrect interpretation of dural thickening on 71 (34.9%) NCCTs, choroid plexus calcifications on 58 (28.6%) NCCTs, and post-surgical changes or intracranial tumors on 24 (11.8%) (Figure 2b,c; Table 2).

## 4. Discussion

Integrating AI-based ICH detection tools into ED workflows aims to enhance the identification of ICH and potentially improve patient care. The primary finding of our retrospective study, performed on a large collection of consecutive NCCTs from patients who were referred from the ED, showed that the sensitivity of 87.3% and specificity of 74% for RAPID ICH were lower than the sensitivity and specificity listed in the FDA clearances for both RAPID ICH (version 1.0) and RAPID ICH (version 3.0) [16,17]. Results from other studies include a sensitivity of 95.6% and specificity of 95.3% found in Heit et al. using RAPID ICH (version 1.0), a sensitivity of 91.9% and specificity of 84.4% in Eldaya et al. with RAPID ICH (version 1.0), a sensitivity of 97.8% and specificity of 99.5% in Sreekrishnan et al. with RAPID ICH (version 3.0) and a sensitivity of 96.2% and specificity of 99.5% in Yedevalli et al. with RAPID ICH (version 3.0) [12,13,19,20]. A systematic review and meta-analysis that assessed various DL models for ICH detection in NCCTS, of which some were commercially available, showed a pooled sensitivity of 92% and a pooled specificity of 94% [10]. Slightly lower results were found in a systematic review that analyzed AI models for ICH detection in NCCTs, which included commercially available tools and some AI models that were not DL-based, with a pooled sensitivity and specificity of 90% and 90% [9]. Our sensitivity and specificity were lower than those in the cited studies. Differences in the percentage of ICH-positive NCCTs included in the cohorts of those studies could account for this variation. When a cohort of consecutive patients with mild traumatic brain injuries was assessed for ICH-positive NCCTs based on radiology reports, the prevalence was 8.5% [21]. Two studies that analyzed two different commercially available automated tools for ICH detection in NCCTs in cohorts of consecutive patients with various emergency indications found prevalences of 9.8% and 10.4% for ICH-positive NCCTs defined by ground-truth labeling [22,23]. The percentage of ICH-positive NCCTs based on ground-truth labeling in our cohort was 7.5%, a prevalence in line with the aforementioned prevalences, indicating an unbalanced collection of ICH-positive and -negative NCCTs and therefore reflecting their expected real-world prevalence. In contrast, the prevalence of ICH-positive NCCTs resulting from ground-truth labeling in Heit et al. and Sreekrishnan et al. was 52% and 52%, showing a balanced collection of ICH-positive and -negative NCCTs, which is not found in real-world consecutive cohorts [12,13]. The most closely comparable results for sensitivity and specificity to our study were reported in Eldaya et al. in a cohort of consecutive patients with suspected stroke, which included 12.1% ICH-positive NCCTs based on ground-truth labeling and consequently also represented an unbalanced collection of ICH-positive and -negative NCCTs [19]. Yedevalli et al. analyzed an unbalanced collection of ICH-positive and -negative NCCTs, with 10.7% of their samples being ICH-positive NCCTs, as determined by ground-truth labeling, but used RAPID ICH (version 3.0), which could be an explanation for the difference in sensitivity and specificity from our study [20].

The secondary finding was a remarkably low PPV from RAPID ICH. The low PPV in our study may be partially attributed to the low prevalence of ICH-positive NCCTs in our cohort, since many previous studies enriched their NCCT collections with ICH-positive NCCTs and therefore inflated the PPV [24]. Another reason for the low PPV is the high number of FP RAPID ICH identifications. The most frequent suspected causes of FP RAPID ICH identifications were non-blood-related hyperintensities like dural thickening or calcifications, findings that have previously been attributed to FP ICH identifications [19,25]. One possible reason for the high number of FP RAPID ICH identifications could be the median age of our patients, since it is known that the incidence of vascular calcifications, dural calcifications, and choroid plexus calcifications increases with age [23,26]. Seven out of the eight FN RAPID ICH identifications were related to small ICHs with a maximum size of circa 6 mm. Similar FN ICH identifications were reported by Heit et al. using RAPID ICH (version 1.0), and it is known that RAPID ICH is limited to detecting suspected ICHs no smaller than 0.4 mL [12,13]. A low PPV coupled with a high number of FP ICH detections increases the burden on radiologists. This additional workload stems from the need to review and rule out numerous incorrect detections, therefore increasing the risk of alert fatigue. In a study that analyzed ICH detection using another commercially available automated tool for ICH detection, the risk of alert fatigue was taken into consideration, even if the automated tool had a PPV of 82% [27].

The first-year radiology resident achieved better results compared to RAPID ICH regarding sensitivity, specificity, and PPV, while RAPID ICH maintained a comparable level for NPV. A prior study demonstrated that a radiology resident surpassed an FDA-approved DL program for ICH detection on NCCT examinations [22]. Another DL tool designed for ICH detection on NCCT examinations, in comparison to junior radiology residents, achieved comparable sensitivity results but exhibited lower specificity [28]. Moreover, when an ICH-detection DL tool was used to assist neuroradiologists, board-certified radiologists, and physicians without training in radiology for the detection of ICH, the greatest improvement in ICH detection was achieved among the group of doctors without training in radiology [29]. Therefore, testing DL tools by comparing them with radiologists of varying experience levels, or looking at the impact that a DL tool has in assisting radiologists and non-radiology physicians in ICH detection, can provide valuable insights into their performance.

Our study has several limitations that have to be addressed. First, this is a single-site retrospective study supported by a large cohort of consecutive ED patients, and the applicability of these findings to other institutions remains uncertain. Second, the usage of a single CT scanner with the same CT protocol may reduce the possibility of applying the results to other types of CT scanners. Third, RAPID ICH (version 3.0) represents the most recent iteration of this DL tool and demonstrates improved sensitivity and specificity for identifying ICH-positive NCCT images compared to RAPID ICH (version 1.0), which was utilized in our study due to its availability during the research period. Having the possibility to examine the same group of NCCTs that were presented in our study with different versions of RAPID ICH would be valuable for the assessment of performance metrics between the versions. Unfortunately, RAPID ICH does not allow the use of different versions concurrently, since it is a commercially available DL tool integrated in a real-world clinical setting. Fourth, it was not possible to explore the performance of RAPID ICH regarding ICH categories, since RAPID ICH reports only the presence or absence of a suspected ICH. Fifth, we excluded NCCT examinations with severe motion or metallic artifacts, which radiologists also find difficult to interpret. Even if this is a common practice in many studies, it could be a source of spectrum bias. Future research should focus more on spectrum bias when externally validating automated ICH detection tools, similar to current practices for DL tools in ischemic stroke analysis [30]. Sixth, we compared the results of only one first-year radiology resident to the results of RAPID ICH for ICH detection. A more appropriate performance analysis at a group level should include more radiology residents with different levels of training. Finally, we did not measure the duration required for RAPID ICH to deliver the notification of suspected ICH detection to the hospital’s PACS, as this is particularly critical for acute patients awaiting therapy decisions.

## 5. Conclusions

RAPID ICH’s low PPV and high number of FP ICH identifications limit the generalizability of the assessed version of this DL tool in similar real-world settings and emphasize the need for further real-world studies with external validation. Implementing commercially automated tools for ICH detection into the hospital workflow should not increase the workload of radiologists; therefore, pre-implementation impact evaluation is essential.

## Figures and Tables

**Table 1:** Sensitivity, specificity, positive predictive value (PPV), negative predictive value (NPV), and area under the curve (AUC) with a 95% confidence interval (CI), as well as the F1 score for RAPID ICH’s identifications against ground-truth labeling, and that for the first-year radiology resident’s ICH identifications against ground-truth labeling.

	RAPID ICH	First-Year Radiology Resident
Sensitivity	87.3% [95% CI: 76.5–94.4%]	95.2% [95% CI: 86.7–99%]
Specificity	74% [95% CI: 70.8–77.1%]	90.8% [95% CI: 88.5–92.7%]
PPV	21.3% [95% CI: 16.5–26.8%]	45.5% [95% CI: 36.8–54.3%]
NPV	98.6% [95% CI: 97.3–99.4%]	99.6% [95% CI: 98.8–99.9%]
AUC	0.81 [95% CI: 0.76–0.85]	0.93 [95% CI: 0.90–0.96]
F1 score	0.34	0.62

**Table 2:** Analysis of false-positive RAPID ICH identifications according to suspected cause.

Discrepancy Type	Suspected Cause of False-Positive RAPID ICH Identification (*n*, %)
False positive (*n* = 203)	Dural thickening—thick falx cerebri or/and thick tentorium cerebelli (74, 34.9%)
	Choroid plexus calcifications (58, 28.6%)
	Post-surgical/tumor (24, 11.8%)
	Other calcified intracranial structures—basal ganglia or/and pineal gland (18, 8.9%)
	Streak or beam hardening artifacts (14, 6.9%)
	Basilar artery or/and middle cerebral artery (12, 5.9%)
	Venous sinus (6, 2.9%)

## Data Availability

The original contributions presented in this study are included in the article. Further inquiries can be directed to the corresponding author.

## Abbreviations

The following abbreviations are used in this manuscript:

NCCT	Non-contrast head computed tomography
ICH	Intracranial hemorrhage
AI	Artificial intelligence
FDA	Food and Drug Administration
CE	Conformité Européene
DL	Deep learning
ML	Machine learning
ED	Emergency Department
FN	False negative
FP	False positive
PPV	Positive predictive value
NPV	Negative predictive value
CI	Confidence interval
AUC	Area under the curve
